# A Comprehensive Review of Artificial Intelligence in Prostate Cancer Care: State-of-the-Art Diagnostic Tools and Future Outlook

**DOI:** 10.7759/cureus.66225

**Published:** 2024-08-05

**Authors:** Somya Agrawal, Sunita Vagha

**Affiliations:** 1 Pathology, Jawaharlal Nehru Medical College, Datta Meghe Institute of Higher Education and Research, Wardha, IND

**Keywords:** healthcare innovation, precision medicine, machine learning, diagnostic tools, artificial intelligence, prostate cancer

## Abstract

Prostate cancer remains a significant global health challenge, characterized by high incidence and substantial morbidity and mortality rates. Early detection is critical for improving patient outcomes, yet current diagnostic methods have limitations in accuracy and reliability. Artificial intelligence (AI) has emerged as a promising tool to address these challenges in prostate cancer care. AI technologies, including machine learning algorithms and advanced imaging techniques, offer potential solutions to enhance diagnostic accuracy, optimize treatment strategies, and personalize patient care. This review explores the current landscape of AI applications in prostate cancer diagnostics, highlighting state-of-the-art tools and their clinical implications. By synthesizing recent advancements and discussing future directions, the review underscores the transformative potential of AI in revolutionizing prostate cancer diagnosis and management. Ultimately, integrating AI into clinical practice can potentially improve outcomes and quality of life for patients affected by prostate cancer.

## Introduction and background

Prostate cancer is a prevalent and significant health concern worldwide, ranking among the most commonly diagnosed cancers in men. Its incidence underscores the urgent need for effective diagnostic and therapeutic strategies [1]. Early detection plays a crucial role in mitigating the impact of prostate cancer by enabling timely intervention, potentially improving patient outcomes and reducing mortality rates. However, current diagnostic methods, such as prostate-specific antigen (PSA) testing and traditional imaging techniques, face challenges such as variability and limitations in accuracy [1].

Artificial intelligence (AI) represents a transformative innovation in healthcare, offering promising solutions to enhance diagnostic precision and efficiency across various medical domains. In prostate cancer diagnostics specifically, AI has the potential to revolutionize how clinicians detect and manage the disease [2]. AI technologies aim to augment human capabilities in interpreting complex medical data by harnessing large datasets and advanced algorithms. This augmentation could lead to earlier and more accurate detection of prostate cancer, thereby facilitating prompt and targeted treatment decisions [3].

This review explores the current landscape of AI applications in prostate cancer care. It will explore state-of-the-art AI-driven diagnostic tools, their clinical implications, and potential future directions. By synthesizing existing knowledge and recent advancements, the review seeks to provide insights into how AI can improve diagnostic accuracy, optimize treatment strategies, and ultimately enhance patient outcomes in prostate cancer care. By critically examining the role of AI in this context, the review aims to inform healthcare professionals, researchers, and policymakers about the transformative potential of AI in prostate cancer diagnostics and management.

## Review

Traditional diagnostic approaches for prostate cancer

Biopsy Techniques and Challenges

Prostate-specific antigen (PSA) testing is commonly used as an initial step in diagnosing prostate cancer. PSA is a serum biomarker that can suggest the presence of prostate cancer, although it is not a definitive diagnostic test. Elevated PSA levels may indicate prostate cancer but can also be caused by other conditions, leading to a high rate of false positives. While PSA testing has facilitated earlier detection of prostate cancer, it has also increased the identification of low-risk cancers that may not require treatment [4]. Digital rectal examination (DRE) is another traditional method for diagnosing prostate cancer. This physical examination of the prostate gland by a physician can suggest the presence of cancer if abnormalities are found. However, DRE is not a reliable diagnostic tool, with studies showing that when performed by primary care physicians, it has a sensitivity of only 0.51 and a specificity of 0.59, reflecting its limited accuracy [5]. Transrectal ultrasound (TRUS) is employed to guide prostate biopsies. However, TRUS is unsuitable as a standalone screening tool due to its high false-positive rate. The TRUS-guided systematic biopsy, which involves taking 12 or more core samples, is commonly used to obtain a tissue diagnosis of prostate cancer. Despite its utility, this method can result in overdiagnosis and overtreatment of low-risk cancers [6]. Multiparametric MRI (mpMRI) has become a valuable tool for detecting and localizing clinically significant prostate cancers. mpMRI integrates T2-weighted, diffusion-weighted, and dynamic contrast-enhanced imaging to provide a comprehensive view of the prostate gland. The NCCN guidelines now recommend mpMRI as the initial diagnostic test for patients with elevated PSA levels or abnormal DRE findings before biopsy. This approach can help reduce the number of unnecessary biopsies and enhance the detection of clinically significant cancers [7].

Current Imaging Modalities and Their Limitations

PSA testing is widely used as a serum biomarker for prostate cancer screening. However, elevated PSA levels can also be caused by non-cancerous conditions, leading to false-positive results. While PSA screening has enabled earlier detection of prostate cancer, it has also increased the identification of low-risk cancers that may not necessitate immediate treatment [4]. DRE involves a physician physically examining the prostate gland. Although DRE can offer helpful information, it has been shown to have limited diagnostic accuracy, with a sensitivity of only 0.51 and a specificity of 0.59. This suggests that DRE when performed by primary care physicians, may not be a reliable standalone diagnostic tool for prostate cancer [8]. TRUS is primarily used to guide prostate biopsies but is not suitable as a standalone screening method due to its high false-positive rate. The TRUS-guided systematic biopsy, which involves collecting 12 or more core samples, is a common technique for obtaining a tissue diagnosis of prostate cancer [9]. mpMRI has proven to be a valuable tool for detecting and localizing clinically significant prostate cancers. mpMRI combines T2-weighted, diffusion-weighted, and dynamic contrast-enhanced imaging to offer a comprehensive assessment of the prostate. Despite its advantages, mpMRI's soft tissue resolution in certain areas may not match that of computed tomography (CT) scans, and MRI scans generally take longer to complete, ranging from 15 minutes to over an hour [10]. CT scans use a series of X-ray images taken from various angles to create detailed cross-sectional images of the body. Although CT scans provide valuable information, they involve exposure to ionizing radiation, and some patients may experience allergic reactions or kidney issues related to the contrast dye used [11].

Prostate-Specific Antigen Testing and Its Role in Screening

The PSA test measures the level of PSA, a protein produced by the prostate gland, in the blood. Elevated PSA levels can suggest the presence of prostate cancer, but the PSA test is not a definitive diagnostic tool. Increased PSA levels may also result from non-cancerous conditions such as prostatitis or benign prostatic hyperplasia (BPH) [4]. While there is no standard or routine screening test for prostate cancer, the PSA test is commonly used for this purpose. The benefits and drawbacks of routine PSA screening are debated, as it can lead to the detection of slow-growing cancers that might not require treatment. Many medical organizations now advocate for an individualized approach to PSA screening, considering the patient's risk factors and preferences. Screening is generally recommended for men aged 55 to 69, while the U.S. Preventive Services Task Force (USPSTF) advises against screening for men aged 70 and older. Men at higher risk, such as those with a family history of prostate cancer or of African descent, may be advised to begin screening earlier, around ages 40 to 45 [12]. PSA tests can yield false-positive results, which may lead to unnecessary biopsies and increased anxiety. Furthermore, differentiating between slow-growing and more aggressive prostate cancers based solely on PSA levels is challenging. Therefore, the PSA test serves a crucial yet complex role in prostate cancer screening, with its benefits and risks needing to be carefully assessed on an individual basis in consultation with a healthcare provider [13].

Role of artificial intelligence in prostate cancer diagnosis

Machine Learning Algorithms

AI has become an increasingly valuable asset in diagnosing and managing prostate cancer. One of the critical applications of AI in this field is the analysis of prostate MRI scans. AI-powered algorithms have shown promising performance in detecting clinically significant prostate cancer on MRI, often outperforming radiologists. These AI systems assist radiologists by enhancing the accuracy of interpretations and reducing the time required for analysis [14]. Another significant application of AI is in automated pathology analysis. AI algorithms support pathologists by automating tasks such as analyzing prostate biopsy samples and determining Gleason scores. This can result in faster, more consistent, and potentially more accurate diagnoses, as AI systems process and analyze samples more efficiently than manual methods [15]. In addition to radiology and pathology, AI has been utilized to optimize various aspects of prostate cancer radiotherapy. AI can automate the calculation of dose distributions, estimate delivered doses, and predict treatment outcomes. This improves radiotherapy planning, delivery efficiency, and precision, leading to better patient outcomes [16]. Although AI's role in prostate cancer surgery is less explored, the technology also holds potential for applications in this area. AI-powered systems could assist surgeons through robotic guidance and intraoperative decision support, enhancing the precision and effectiveness of surgical interventions [17]. As AI continues to be integrated into prostate cancer care, its future outlook is promising. Advancements in AI-based prostate MRI analysis, pathology automation, and radiotherapy planning are expected to improve diagnostic accuracy, treatment planning, and patient outcomes. However, the adoption of AI also faces challenges, including ensuring the clinical validity and safety of AI algorithms, addressing data privacy concerns, and integrating the technology into existing clinical workflows [14].

Natural Language Processing

Extracting insights from clinical notes and pathology reports is crucial but challenging in modern healthcare. These documents are often complex and contain dense medical jargon and abbreviations that can be difficult for natural language processing (NLP) systems to interpret. Variations in language, negation, and human error across different reports further complicate the generalization of NLP models. Additionally, scanned or handwritten reports introduce another layer of complexity compared to structured electronic health record data [18]. Advanced NLP techniques are being developed to address these challenges. Named entity recognition (NER) models, such as BERT, can accurately extract detailed diagnostic information from pathology reports. Data augmentation methods enhance the robustness of NLP models, enabling them to handle variations in language across diverse medical reports. Automated extraction of pathological features from scanned reports can significantly reduce the manual effort required for medical research compared to traditional data abstraction methods [19]. The insights extracted from clinical notes and pathology reports can support various medical research, from disease-related studies to clinical workflow optimization. NLP-powered information extraction facilitates the secondary use of electronic health record data buried in unstructured text. Enhanced and visually accessible "smart reports" can make complex pathology test results more understandable for patients, allowing them to be more actively involved in their healthcare decisions. Overall, advancements in NLP techniques are improving the accuracy and robustness of information extraction from clinical notes and pathology reports, with significant implications for supporting medical research, enhancing patient understanding, and maximizing the value of electronic health record data [20].

State-of-the-art artificial intelligence tools in prostate cancer imaging

Radiomics and Texture Analysis

Quantitative imaging biomarkers (QIBs) are measurable features extracted from medical images that offer valuable insights into tumor characteristics beyond what is visible to the naked eye. Radiomics involves the high-throughput extraction of quantitative imaging features related to tumor intensity, texture, shape, and size. Texture analysis techniques, such as the gray-level co-occurrence matrix (GLCM), capture pixel intensity patterns, revealing tumor heterogeneity information. Machine learning algorithms applied to radiomics features can enhance accuracy in distinguishing benign from malignant lesions and predicting clinically significant cancer [21]. QIBs show promise for predicting and monitoring cancer immunotherapy responses, which rely on the tumor microenvironment. Radiomics-based AI and molecular imaging are the most promising diagnostic technologies for immunotherapy assessment. For instance, imaging features such as a low pre-treatment apparent diffusion coefficient (ADC) and a significant reduction in permeability-related enhancement (PE) on MRI have been linked to immune-activated tumor microenvironments in HER2+ breast cancer [22]. The successful clinical translation of QIBs depends on the robustness of measurements across various image processing factors, such as segmentation methods. Reducing errors associated with segmentation is crucial to ensuring the reproducibility of QIB measurements and their associations with clinical outcomes. Integrating QIBs with other non-invasive methods, such as liquid profiling, could facilitate more personalized cancer treatment decisions [23].

Deep Learning Models

Neural networks have demonstrated significant potential in image interpretation and feature extraction. Hierarchical neural network architectures are designed to emulate the robustness and efficiency of human perception in image analysis. These architectures can be trained using unsupervised and supervised learning techniques to tackle various computer vision tasks [24]. In deep neural networks, the features learned progress from simple to more complex as one moves deeper into the network. Convolutional neural networks (CNNs) are particularly effective for image processing tasks, utilizing specialized layers such as convolution and pooling to handle spatial information in images efficiently. The initial layers of a CNN capture low-level features like edges and shapes, while the deeper layers identify more complex and abstract features [25]. Techniques such as activation maximization can visualize the specific features that individual neurons in a CNN are tuned to detect, offering insight into the network's internal representations. CNNs have shown promising results in medical imaging applications, such as detecting anomalies in chest X-rays. Their ability to automatically learn relevant features from large datasets makes them highly effective in assisting radiologists with medical image analysis [26].

Artificial intelligence-driven biomarkers and predictive models

Genomic Data Integration

Integrating AI with genomic profiling advances the precision of risk stratification, treatment selection, and outcome prediction for prostate cancer patients, moving toward a more personalized approach to care. AI algorithms are employed to develop novel biomarkers from multi-omic data, combining digital pathology, clinical, and genomic information. These AI-driven biomarkers offer more accurate outcomes and treatment response predictions than traditional risk stratification methods [16]. Through advanced computational techniques, researchers have discovered new molecular subtypes and therapeutic targets in prostate cancer by integrating diverse data types such as genomics, transcriptomics, metabolomics, and clinical data. This integration supports more personalized treatment strategies. AI models that leverage genomic and other data have shown potential in predicting which prostate cancer patients are likely to benefit from specific treatments, such as ADT or radiation therapy, thus facilitating tailored treatment plans [27]. Moreover, AI-powered tools can automate the processing and analysis of extensive genomic datasets, making it more feasible to incorporate genomic profiling into routine clinical care for prostate cancer patients. This capability accelerates the translation of genomic insights into personalized medicine. However, challenges related to data quality, algorithm transparency, and clinical implementation must be addressed to fully realize the potential of this approach [28].

Risk Assessment Models

AI-driven predictive models have demonstrated significant potential in evaluating prognosis and treatment response for prostate cancer patients. For instance, researchers have developed an AI-derived biomarker capable of identifying men with high-risk localized prostate cancer who could potentially avoid long-term hormone therapy (ADT) without sacrificing efficacy. This AI biomarker, trained on data from multiple clinical trials, predicts which patients would benefit most from prolonged ADT and which could be managed with a shorter duration, thus reducing unnecessary side effects [29]. Multi-modal AI (MMAI) systems, which integrate digital histopathology with clinical data, have led to the development of prognostic models that surpass the standard NCCN risk groups for localized prostate cancer. This MMAI approach has been adapted to create a predictive model for identifying patients likely to benefit differentially from adding ADT to radiation therapy. Additionally, AI models have demonstrated high accuracy in predicting lymph node metastasis in patients with oral cavity cancer, a crucial prognostic factor in head and neck cancers. These AI-powered tools could greatly aid clinicians in treatment planning and follow-up for prostate cancer patients [30]. Integrating AI-driven predictive models is promising to enhance prostate cancer care by improving treatment selection and personalization and streamlining clinical workflows and decision-making. Nonetheless, challenges related to algorithm transparency, data bias, and clinical integration persist, necessitating continued collaboration among clinicians, researchers, and technology providers [31].

Clinical applications and validation studies

Review of Clinical Trials and Real-World Applications

In one clinical trial, researchers assessed an AI-derived digital pathology biomarker designed to identify men with high-risk localized prostate cancer who could potentially avoid long-term hormone therapy (androgen deprivation therapy or ADT) without compromising efficacy. This AI biomarker, developed from data across multiple clinical trials, successfully predicted which patients would benefit from extended ADT and which could be managed with a shorter duration, thereby minimizing unnecessary side effects [29]. Another study employed an MMAI system that integrated digital histopathology with clinical data to develop and validate prognostic models. These models outperformed traditional NCCN risk groups for localized prostate cancer. The MMAI approach was further adapted to create a predictive model for identifying patients likely to benefit from adding ADT to radiation therapy [29]. In practical settings, AI systems are now assisting radiologists with interpreting prostate MRI scans. These algorithms automate tasks such as lesion segmentation and risk scoring, enhancing efficiency and consistency in MRI analysis. AI-powered algorithms have also been incorporated into clinical workflows to analyze pathology slides, accurately identify cancerous regions, and support pathologists in prostate cancer diagnosis and grading [32]. Additionally, AI has been utilized to optimize various aspects of prostate cancer treatment planning, including automated dose distribution calculations for radiation therapy and support for brachytherapy procedures. These AI-powered tools can streamline workflows and improve treatment outcomes [33]. These clinical trials and real-world applications highlight the potential of AI to advance prostate cancer care by enhancing diagnostic accuracy, treatment planning, and personalized treatment recommendations. However, continued research and validation are essential to fully realize the potential of AI in this field [34].

Comparative Studies Between Artificial Intelligence-Driven Diagnostics and Traditional Methods

AI has demonstrated significant promise in advancing prostate cancer diagnostics. AI-driven tools show performance rivaling or surpassing traditional diagnostic methods in several critical areas. Comparative studies underscore the potential benefits of AI-based solutions, including enhanced diagnostic accuracy, cost-effectiveness, accessibility, and workflow optimization [14]. In prostate MRI interpretation, AI systems have proven capable of detecting clinically significant prostate cancer with accuracy comparable to or exceeding that of expert radiologists. These AI algorithms can automate lesion segmentation and risk-scoring processes, streamlining the diagnostic process and enhancing consistency. Similarly, in pathology image analysis, AI-powered tools have shown the ability to analyze slides and identify cancerous regions with high precision, complementing human pathologists' expertise. Integrating data from various sources such as MRI, pathology, and clinical records models offers more comprehensive risk assessments and personalized treatment recommendations than traditional methods [35]. The cost-effectiveness and accessibility of AI-driven diagnostics are notable advantages. AI-based solutions are considered "cost-effective" and offer "affordable, accessible, and accurate alternatives" to traditional diagnostics, particularly in resource-limited settings. Additionally, AI's workflow optimization capabilities can significantly enhance efficiency by automating tasks and improving consistency [36]. However, the integration of AI into healthcare comes with challenges. Developing robust and generalizable AI models requires access to large, diverse, and high-quality datasets, which can be a substantial obstacle. Ensuring the transparency and interpretability of AI-driven diagnostic tools is essential for building trust and facilitating clinical adoption. Furthermore, navigating regulatory and ethical considerations is crucial to safeguard patient safety and privacy [37]. Despite these challenges, the potential benefits of AI-driven diagnostics in prostate cancer care are substantial. Continued research and collaboration among clinicians, researchers, and technology providers will be crucial in fully realizing AI's potential, ultimately leading to improved patient outcomes and more efficient healthcare delivery [28].

Challenges and limitations

Regulatory Challenges and Approval Processes

Integrating AI into prostate cancer care can enhance clinical decision-making, reduce documentation burden, and improve patient outcomes. One prominent area of AI advancement is medical image analysis for prostate cancer detection and diagnosis [28]. Studies have shown that AI systems can accurately detect clinically significant prostate cancer on MRI scans, often matching or exceeding radiologists' performance. AI algorithms are proficient at identifying suspicious lesions and differentiating between significant and insignificant prostate cancer. Beyond imaging, AI is also transforming pathology sample analysis. AI-powered tools can aid pathologists by detecting and grading prostate cancer in biopsies, potentially improving consistency and reducing turnaround times [14]. AI is expected to increasingly influence various aspects of prostate cancer management, including treatment planning, outcome prediction, and optimization of radiation therapy dosage plans. Researchers are also exploring AI's potential to predict treatment toxicity and determine the most effective surgical approaches. Despite these advancements, the widespread adoption of AI in prostate cancer care faces challenges such as the need for rigorous clinical validation, data privacy and security concerns, and the risk of bias in AI algorithms. Addressing these issues through continued research and collaboration among clinicians, researchers, and technology providers is essential for realizing AI’s full potential in prostate cancer care [17]. Integrating AI-driven biomarkers and predictive models holds promise for improving treatment selection, personalizing care, and streamlining clinical workflows. However, challenges such as bias, lack of standardization, data quality and heterogeneity, and reproducibility issues must be addressed. Ensuring rigorous validation, safeguarding data privacy and security, and achieving seamless integration into clinical workflows are crucial for the successful implementation of AI in prostate cancer care [38].

Ethical Considerations in Artificial Intelligence-Driven Diagnostics

Ethical considerations in AI-driven diagnostics for prostate cancer care are paramount and require careful attention. One primary concern is the potential for AI algorithms to perpetuate existing societal biases, which could result in suboptimal care for certain socioeconomic groups if the training data lacks representativeness and diversity. Developers of AI tools must actively identify and mitigate such biases to prevent exacerbating health disparities [39]. Data privacy and security are critical issues as well. Implementing robust safeguards to protect sensitive patient data used to train AI systems is essential. Transparency in data collection, handling, and storage practices is necessary to build and maintain trust in these technologies [40]. Accountability and explainability are also critical ethical considerations. Clinicians must be able to justify using AI-driven outputs in decision-making processes, which underscores the need for explainable AI systems. Ensuring transparency and fairness in AI algorithms is crucial for fostering trust and acceptance in AI-driven healthcare [41]. Regulatory oversight is another area that demands careful attention. Existing ethical frameworks and regulatory guidelines may need to be adapted to address the specific challenges posed by AI-driven diagnostics. Detailed frameworks for the approval and monitoring of AI systems in healthcare are currently lacking and must be developed by policymakers [42]. Finally, the role and responsibility of clinicians in utilizing AI tools must be clearly defined. While AI is not expected to replace clinical judgment entirely, clinicians who use these technologies must assume ultimate responsibility for their outcomes. Ensuring clinicians receive proper training and education on AI systems' practical and ethical use is essential [42]. Proactively addressing these ethical considerations will be crucial for harnessing the full potential of AI in prostate cancer diagnostics while adhering to fundamental principles of patient care. Ongoing collaboration among technologists, clinicians, ethicists, and policymakers will be necessary to navigate these complex challenges [42].

Integration With Existing Healthcare Infrastructure

Integrating AI into existing healthcare infrastructure presents several challenges that must be addressed for effective implementation. A vital issue is interoperability, as AI systems often need to share data across diverse platforms and systems. Ensuring secure data transfer while preserving integrity and confidentiality is vital. This requires adopting standardized formats and protocols and fostering collaboration between technology vendors and healthcare organizations to develop interoperable solutions [43]. Another challenge is integrating AI-driven technologies with existing legacy healthcare systems, which compatibility issues can complicate. Significant investments may be necessary to upgrade or replace outdated infrastructure to facilitate seamless AI integration. In countries like India, where healthcare infrastructure is uneven and gaps exist in remote and underserved areas, implementing AI-driven solutions such as telemedicine can be particularly challenging due to limited connectivity and technological resources [44]. AI systems depend heavily on high-quality data, but healthcare organizations may encounter issues with insufficient or poor-quality data for training AI algorithms. Strategies are needed to collect, store, and maintain high-quality data alongside fostering data-sharing collaborations. Additionally, healthcare organizations must adhere to stringent regulations, such as HIPAA, to protect patient privacy. Integrating AI while ensuring regulatory compliance is complex, necessitating robust data security measures, transparency in AI decision-making, and accountability for AI-driven outcomes [45]. Finally, healthcare professionals may be reluctant to adopt AI-driven technologies, requiring education and a cultural shift toward embracing these innovations. Addressing these challenges through strategic planning, infrastructure improvements, data management, regulatory compliance, and change management will be crucial for successfully integrating AI into healthcare systems [46].

Future directions and outlook

Emerging Trends in Artificial Intelligence for Prostate Cancer Care

**Figure 1:**
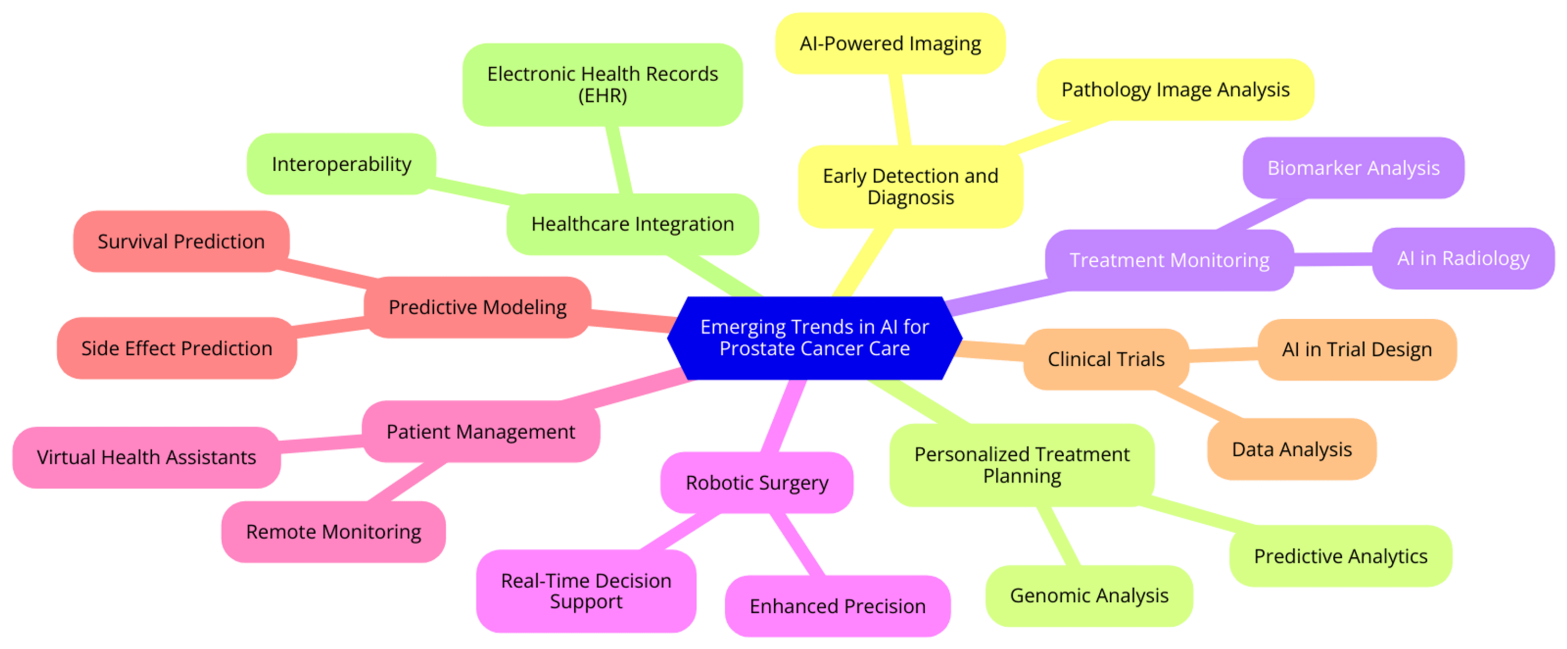
Emerging trends in AI for prostate cancer care AI, artificial intelligence Image Credit: Dr Soumya Agrawal

AI in prostate cancer care is an evolving field with significant potential to enhance diagnosis, treatment planning, and clinical workflows. A major trend is the development of AI algorithms capable of analyzing MRI scans to detect clinically significant prostate cancer with high accuracy. This advancement could reduce the need for invasive biopsies and provide more reliable diagnoses. AI-based software is also being developed to automate the analysis of prostate MRIs, which can help minimize variability in interpretation among radiologists [34]. Another key trend is the application of AI to facilitate more personalized treatment approaches for prostate cancer. In radiotherapy, AI can automate dose calculations, optimize treatment plans, and predict outcomes such as the risk of toxicity. In brachytherapy, where radioactive sources are implanted into the prostate, AI can assist with imaging analysis and treatment planning to enhance efficiency and consistency. One study utilized multi-modal deep learning on clinical trial data to tailor prostate cancer therapy based on individual patient profiles [47]. AI also has the potential to significantly streamline various aspects of prostate cancer care. By automating tasks such as prostate MRI interpretation and treatment planning, AI can reduce the time and effort required while providing decision support to clinicians to improve consistency and quality of care. Integrating AI into clinical workflows will be critical to achieving these efficiency gains [3]. Despite these advances, several challenges and limitations need to be addressed. Ensuring that AI algorithms are well-validated, unbiased, and generalizable to diverse patient populations is crucial. Integrating AI into clinical workflows in a manner that enhances rather than disrupts patient care is also a significant concern. Additionally, regulatory, ethical, and data privacy issues surrounding the use of AI in healthcare must be carefully considered [48]. Emerging trends in AI for prostate cancer care are shown in Figure 1.

Potential Impact on Patient Outcomes and Healthcare Efficiency

The impact of AI on patient outcomes and healthcare efficiency is profound. AI systems can analyze medical images, such as MRI scans and pathology slides, with exceptional precision, detecting subtle abnormalities that human clinicians might overlook. This capability can facilitate earlier diagnosis and intervention, thereby enhancing patient outcomes. AI-powered predictive analytics can foresee health risks and potential complications before they become critical, enabling proactive care and prevention. Moreover, AI can streamline administrative tasks such as scheduling and bed management, allowing healthcare providers to concentrate more on patient-centric care. AI-driven clinical decision support tools can augment clinicians' capabilities by alleviating the burden of analyzing test results and medical images [49]. AI can further boost patient engagement and adherence by delivering personalized guidance, medication reminders, and remote monitoring. AI-powered virtual health assistants can offer tailored interventions and "nudge" patient behavior by comparing individual data with effective treatment pathways from similar patient cohorts. This approach can enhance treatment outcomes and reduce healthcare costs. Additionally, AI-enabled telemedicine and virtual assistants can improve access to healthcare services, particularly in underserved or remote areas. AI-powered diagnostic tools can be deployed in resource-constrained settings to extend the reach of healthcare services. Overall, the integration of AI into healthcare holds the promise of revolutionizing care delivery, enhancing patient outcomes, and improving healthcare efficiency while broadening access to care. Nonetheless, achieving successful integration will necessitate overcoming challenges related to data quality, regulatory approval, and workforce adaptation [50].

*Predictions for the Next Decade in Artificial intelligence*​​​​​​​*-Enhanced Prostate Cancer Diagnostics*

The future of AI-enhanced prostate cancer diagnostics is indeed promising. Over the next decade, we can expect advancements in AI algorithms designed to detect clinically significant prostate cancer on MRI with increased accuracy and efficiency. These systems will likely support radiologists by providing automated lesion detection and analysis, which can help reduce interpretation variability and enhance overall diagnostic precision [51]. Integrating AI into clinical workflows will present challenges, including ensuring data privacy and security and navigating regulatory and ethical concerns. As AI becomes more embedded in prostate cancer care, it will be crucial to address these issues to ensure successful implementation. Enhanced collaboration between countries and institutions will be vital to advancing AI-powered diagnostics and overcoming these hurdles [52]. Future research will likely focus on improving noninvasive diagnostic methods and refining minimally invasive treatments using deep learning technologies. AI has the potential to revolutionize prostate cancer care by enhancing diagnostic accuracy, optimizing treatment planning, and improving patient outcomes. Despite these exciting prospects, careful attention to implementation and regulatory challenges will be necessary to fully realize AI's transformative potential in prostate cancer diagnostics [53].

## Conclusions

In conclusion, integrating AI into prostate cancer care represents a promising frontier in medical innovation. AI-powered diagnostic tools have shown the potential to enhance the accuracy and efficiency of prostate cancer detection, offering clinicians valuable insights from complex medical data that could lead to earlier interventions and improved patient outcomes. While challenges such as regulatory hurdles and ethical considerations remain, the rapid evolution of AI technologies suggests a bright future for their application in enhancing diagnostic capabilities and treatment decisions in prostate cancer. As research continues to advance and AI algorithms become more sophisticated, the collaborative efforts of healthcare professionals, researchers, and technology developers will be crucial in realizing the full potential of AI to transform prostate cancer care and improve the lives of patients worldwide.
